# Isolation and Gain Improvement of a Rectangular Notch UWB-MIMO Antenna

**DOI:** 10.3390/s22041460

**Published:** 2022-02-14

**Authors:** Anees Abbas, Niamat Hussain, Md. Abu Sufian, Jinkyu Jung, Sang Myeong Park, Nam Kim

**Affiliations:** Department of Information and Communication Engineering, Chungbuk National University, Cheongju 28644, Korea; anees@cbnu.ac.kr (A.A.); hussain@chungbuk.ac.kr (N.H.); sufian@osp.chungbuk.ac.kr (M.A.S.); yoona106@chungbuk.ac.kr (J.J.); smpark11@korea.kr (S.M.P.)

**Keywords:** rectangular notch, UWB antenna, UWB-MIMO antenna, decoupling structure, mutual coupling

## Abstract

This paper presents the performance improvement of a co-planar waveguide rectangularly notched UWB-MIMO antenna. The isolation and gain of the antenna are enhanced by using a parasitic isolator. The antenna consists of four microstrip patch antennas and an isolator. The UWB characteristic of the antenna is achieved by truncating the lower ends of the radiating patch by a semicircle. The rectangular notch characteristic is obtained by adding two electromagnetic bandgap structures on the backside of the antenna, which is attached to the radiator via shorting pin. The performance, especially the decoupling of the MIMO antenna is improved by using a novel parasitic decoupler, which is placed between the antennas to receive uncorrelated signals. The decoupling structure consists of a square shape metallic element with a circular slot inside and a half-semicircle slot edged at each corner. Four rectangular metallic stubs are extended from opposite parallel sides to improve further isolation. The simulated and measured results show that the antenna has a rectangular notch band (5.25–5.85 GHz) across the working band of 3–12.8 GHz. In addition, the antenna has a planar structure with an overall size of 60 × 60 × 1.52 mm^3^ and offers stable gain, reduced mutual coupling (<−21 dB), and lower envelop correlation (<0.001).

## 1. Introduction

In radio communication, an antenna is an interface between radio waves propagating through space and electric currents moving in metal conductors, used as transmitters or receivers. Because of the simple geometry and low fabrication cost, microstrip technology is a mature and known technology in manufacturing and electromagnetic devices. Hence, it is widely used in designing various microwave and electromagnetic devices such as filters, power dividers, and artificial magnetic surfaces [1,2,3].

Since the Federal Communication Commission (FCC) has set 3.1–10.6 GHz as ultrawideband technology, this has attracted the interest of researchers by offering features like short-range, high bandwidth, low energy usage, low complexity, and high data rate communications [4]. The applications of UWB include short-range communications, sensor networks, tracking, and positioning systems [5,6]. In the multi-path environment, signal fading degrades the performance of UWB systems, which affects the efficiency and quality of signal transmission. To address this problem, Multi-Input-Multi-Output (MIMO) technology has been used to provide a multi-element patch antenna for signal transmission or reception. It can significantly increase spectral efficiency, reliability, and channel capacity without utilizing extra power and spectrum. Hence it is widely used in the design of antennas in different networks to enhance the performance of communication systems. However, the usage of more than one radiating element, closely arranged to one another in MIMO degrades the performance of the system. Therefore, the design of a MIMO system with better isolation is required in modern communications. The UWB-MIMO design with low isolation by keeping a minimum distance between antennas is challenging. So far, different isolation techniques have been used by researchers to reduce mutual coupling in the MIMO antennas [7,8].

These techniques are, defected ground structure [9,10], decoupling network [11,12], space diversity [13], parasitic elements [14,15], neutralization rings [16], different stub and slots [17,18,19,20,21,22], shorting pins [23], metamaterials [24], and non-uniform transmission line using nonlinear model predictive to reduce mutual coupling [25].

The defective ground systems have been widely used in the literature to improve isolation [9,10]. Moreover, a T-shaped ground stub is used in [11] and a strip is placed beneath the radiation patch which is connected through via serving as a decoupler to improve isolation [12]. In addition, with the help of polarization and space diversity, electromagnetic interference has been minimized in [13]. Amin et al. have recommended various parasitic structures to ensure minimal mutual coupling in a 4-port MIMO system [14]. The isolation in [15] is achieved by using parasitic strips and microstrip-fed lines perpendicular to each other. Furthermore, the neutralization line technique is used to solve deterioration in the performance in the MIMO antenna system [16]. Furthermore, to produce high isolation between MIMO antenna elements F-shaped stubs are introduced in the ground plane in [17], and a pair of L-shaped slots are introduced in the feed line for good agreements of isolation in [18]. In [19,20,21,22] high isolation is realized by using slotted stubs decoupling structure. The mutual coupling between antenna elements of the MIMO system is kept down by utilizing different types of metamaterials configurations [24].

In this paper, the performance enhancement of a co-planar waveguide fed (CPW) rectangular notched 2 × 2 UWB–MIMO antenna using a new decoupling structure is presented. The antenna provides good impedance matching from 3–12.8 GHz with a rectangular notch at WLAN-band (5.25–5.85 GHz). We use four antenna elements, which are arranged orthogonally to achieve spatial and pattern diversity. The proposed MIMO antenna follows all MIMO system characteristics with low mutual coupling, reduced ECC, and high diversity gain

## 2. Design Procedure of New UWB-MIMO Antenna

In this section, the UWB MIMO antenna design procedure is discussed step by step. At first, we explain the single element antenna and then, the proposed UWB-MIMO antenna.

### 2.1. Single-Element UWB Antenna

Initially, a simple patch antenna is designed which has a very narrow bandwidth, then the lower end of the patch is truncated with a semicircle to achieve UWB antenna bandwidth. The WLAN band is rejected with high selectivity using a pair of EBG structures [26]. The notched band and frequency can be shifted to the desired interfering bands and frequencies to avoid all kinds of interferences [27].

**Figure 1:**
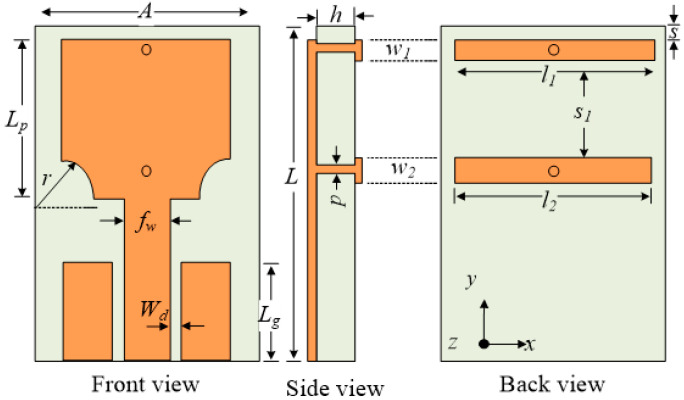
The geometry of the single element UWB antenna [27].

**Figure 2:**
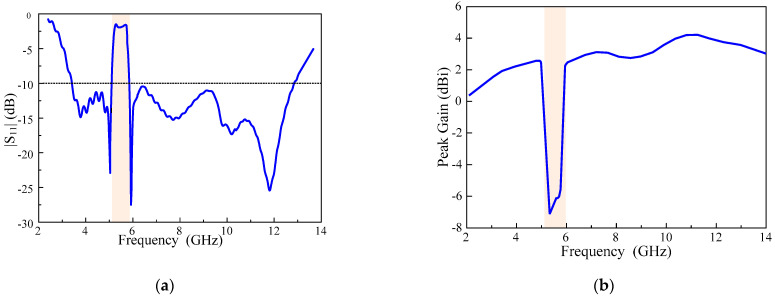
The single element characteristics (**a**) |S_11_| characteristics and (**b**) gain.

The single-element antenna is shown in Figure 1. The antenna has an operational bandwidth of 3–12.8 GHz for |S_11_| < −10 rejecting WLAN band signals rectangularly with high selectivity. The size of the single element antenna is 16 × 25 × 1.52 mm^3^ and it has stable gain and radiation patterns. The impedance characteristics of the antenna in terms of |S_11_| and gain are shown in Figure 2. The gain curve is stable with the maximum value of 4.5 dBi until rejected band where it sharply decreased to about −6 dBi.

### 2.2. UWB-MIMO Antenna without Decoupling Structure

**Figure 3:**
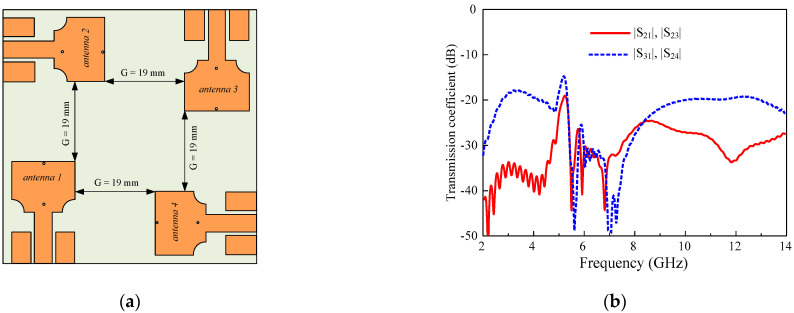
MIMO antenna without decoupling structure (**a**) geometry (**b**) transmission coefficient.

The low mutual coupling and port isolation are crucial for good MIMO performance due to the very small distance between antenna elements. The phenomena of wave propagation inside substrate as well as in the near field of antenna share the overall coupling effect of the MIMO system. In the early stage of the 2 × 2 MIMO design, four elements are placed orthogonally without any decoupling structure to each other as shown in Figure 3a. The distance between antenna elements is kept as minimum as possible to realize the compactness of the MIMO system. The performance of the antenna in this scenario without any decoupling mechanism is simulated. A high coupling (−11 dB) is observed, because of inducting behavior of conducting materials due to nearby current exciting elements as shown in Figure 3b.

### 2.3. UWB-MIMO Antenna with Square Shaped Decoupler

**Figure 4:**
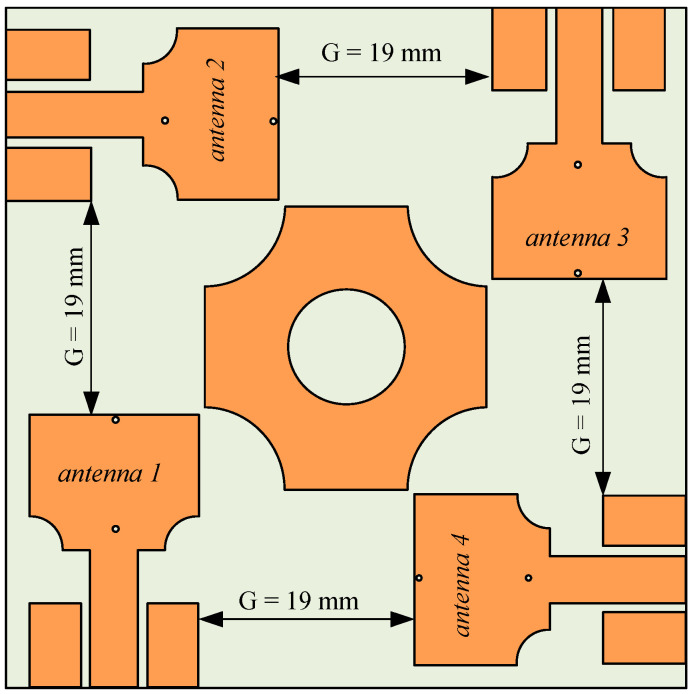
The geometry of the UWB-MIMO antenna with square-shaped decoupler.

**Figure 5:**
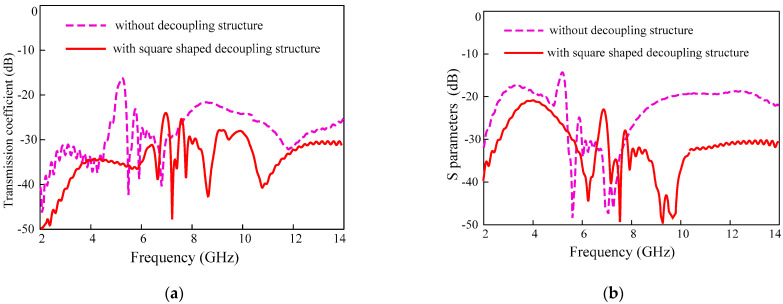
Transmission coefficient for UWB-MIMO antenna with and without square-shaped decoupler (**a**) adjacent antennas (|S_12_|, |S_14_|, |S_23_|, |S_34_|) and (**b**) diagonally located antennas (|S_13_|, and |S_24_|).

The design process of the decoupling structure started with square shape metallic patch in the center of the substrate which is diagonally centered for all four elements of this MIMO antenna. In the center of the square shape isolator, a circle is etched, and all four corners truncated by half-semicircles shown in Figure 4. The simulation results show that this design reduces the mutual coupling between elements located diagonally shown in Figure 5. It can be seen that the reflection coefficient for the adjacent antennas (|S_21_|, |S_23_|) did not change significantly, however, it is improved for the diagonally located antennas (|S_31_|, |S_24_|). Somehow, this design does not reduce the mutual coupling caused due to adjacent elements efficiently. Therefore, the design of the effective parasitic isolator is required to improve the |S_21_| and |S_23_|.

### 2.4. Proposed UWB-MIMO Antenna

**Figure 6:**
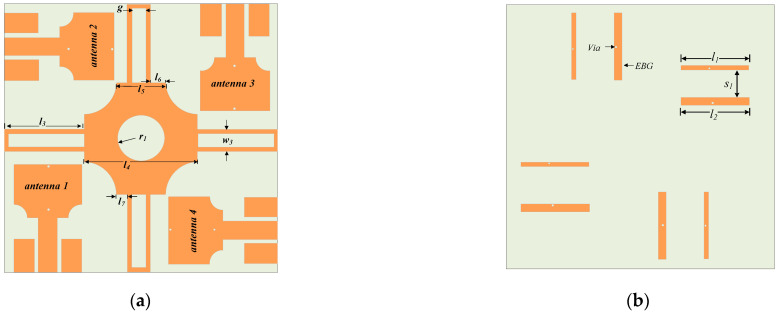
The geometry of the proposed MIMO antenna. (**a**) Front side (**b**) backside.

**Figure 7:**
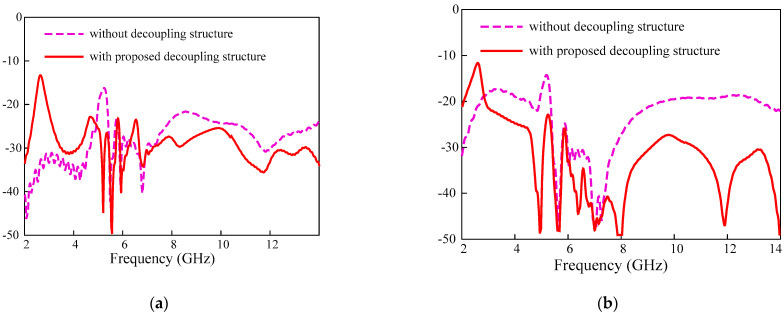
Transmission coefficient for the UWB-MIMO with and without proposed decoupling structure (**a**) adjacent antennas (|S_12_|, |S_14_|, |S_23_|, |S_34_|) and (**b**) diagonally located antennas (|S_13_|, and |S_24_|.

For further isolation improvement in the UWB-MIMO, especially for the adjacent antennas, four rectangular metallic stubs extended from opposite parallel sides of the square shape metal are placed between adjacent antennas. The metallic stubs are connected to square metallic metal which completes the decoupling structure shown in Figure 6. The optimized parameter of the proposed antenna is shown in Table 1. The isolation improvement due to the proposed decoupling structure is shown in Figure 7. The transmission coefficient for the adjacent antennas for all antennas is less than −21 dB in the entire UWB bandwidth.

**Figure 8:**
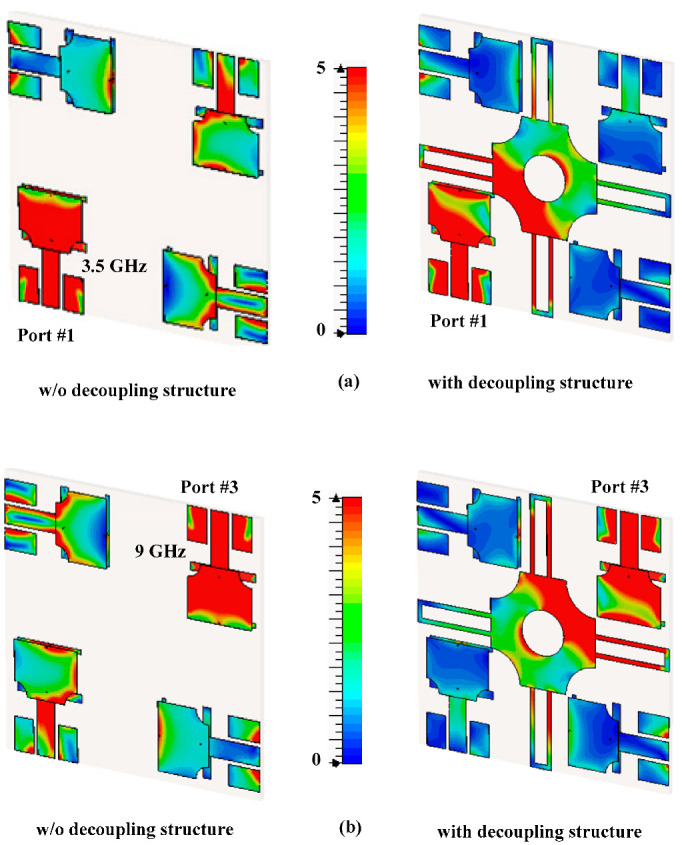
Surface current distribution with and without decoupling structure of different port excitation at (**a**) port-1 and (**b**) port-3.

To understand the electromagnetic behavior of antenna elements and decoupling structure, the surface current distribution for both adjacent and diagonally placed elements is studied. The effectiveness of the decoupling structure can be observed from Figure 8a in which a current at frequency 3.5 GHz has been shown with and without decoupling structure for port 1. While Figure 8b shows the current at frequency 9 GHz with and without decoupling structure for port 3. It can be seen that without decoupling structure, the non-exciting ports also show current which is because of inducting behavior of conducting materials due to nearby current exciting elements. While after introducing a decoupling structure in between these elements, the non-exciting element does not show current flow, because the decoupling structure minimizes the current flow towards others.

## 3. Results and Discussion

**Figure 9:**
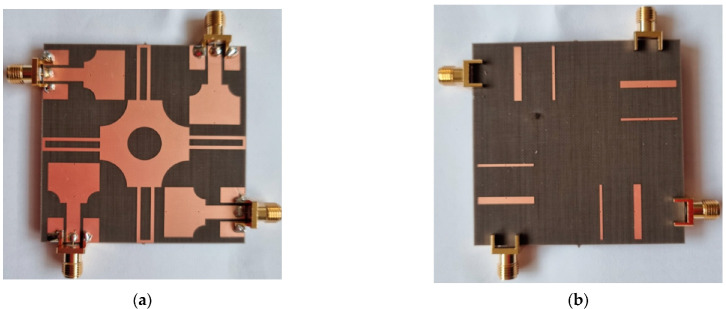
Fabricated proposed antenna: (**a**) Front view (**b**) backside view.

**Figure 10:**
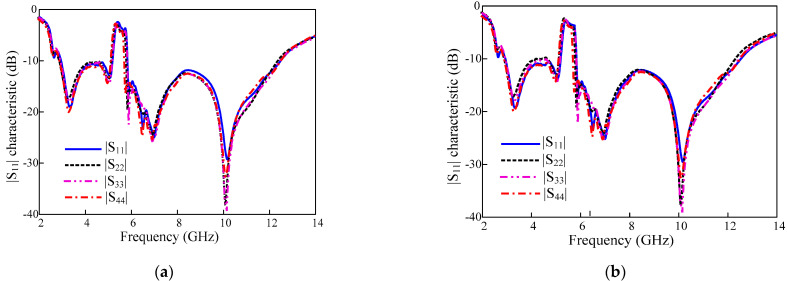
Reflection coefficient of proposed MIMO antenna (**a**) Simulated (**b**) measured.

The proposed antenna was fabricated on Taconic TLY 5 lossy (thickness = 1.52 mm) (εr = 3.55 and tanδ = 0.0009) for experimental verification, which is shown in Figure 9. The impedance and radiation characteristics of the designed antenna are measured by an Agilent vector network. To measure the peak gains of the antenna, a well-calibrated standard horn antenna was used as the source antenna while the fabricated prototype was measured as the receiving antenna. The antenna under test was rotated to measure the peak gain at different orientations. To supply a stable power reception, transmit and receiving amplifiers are used. A stable peak gain curve is obtained in the frequency range of interest with a maximum value of 6.94 dBi, but it sharply decreased at the WLAN band which is filtered. This demonstrates the effectiveness of the antenna’s rectangular notching characteristics. The simulated and measured values of |S_11_|, |S_22_|, |S_33_| and, |S_44_| are compared in Figure 10. Both the simulated and measured results show similar behavior.

The entire UWB spectrum (3–12.8 GHz) shows good impedance matching except for the notched band. The proposed fabricated antenna under measurement was rotating to measure the values of peak gain at different orientations. The simulated and measured gains of the antenna elements at 3 GHz, 5 GHz, 7 GHz, and 9 GHz are shown in Table 2. During the measurement, only one port excited the remaining ports are terminated with a 50-Ω load. The peak gain in the MIMO antenna is 6.94 dBi, increased by 2.44 dBi than a single element which is 4.5 dBi. This increase of the gain in the MIMO antenna system is due to the current redistribution, of more than one radiation element. In literature, there are various techniques used to enhance the gain of antennas such as using metasurface in the near-field of the antenna, additive manufacturing, and more [28,29,30,31,32,33]. However, this work utilizes a parasitic isolator for isolation as well as gain improvements.

### 3.1. Reflection Coefficients

The measured and simulated results of reflection coefficient plots of the MIMO antenna have been shown in Figure 10. The placement of MIMO elements and antenna elements are symmetrical in geometry; therefore, the antennas show the same reflection coefficients.

The MIMO antennas have a similar UWB bandwidth with the WLAN band-notched proposed for the single-element antenna.

### 3.2. Transmission Coefficients

**Figure 11:**
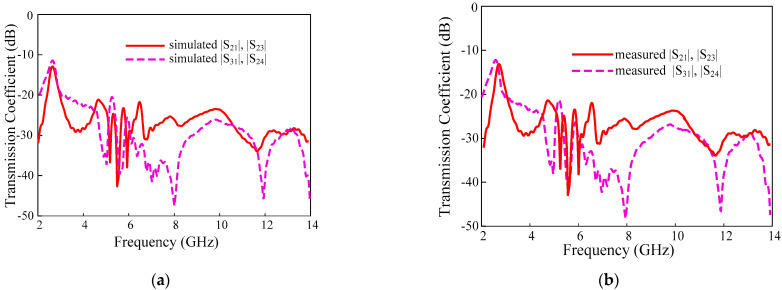
The transmission coefficient of the proposed UWB-MIMO antenna (**a**) simulation and (**b**) measurement.

In MIMO systems, the mutual coupling between the single elements can be expressed as a transmission coefficient. The measured and simulated results are compared in Figure 11. The antenna elements have high isolation characteristics without the proposed decoupling structure.

The proposed parasitic decoupling structure has minimized the isolation of the antennas to >21 dB and the maximum isolation is 50 dB in the entire operating bandwidth.

### 3.3. Envelope Correlation Coefficient

**Figure 12:**
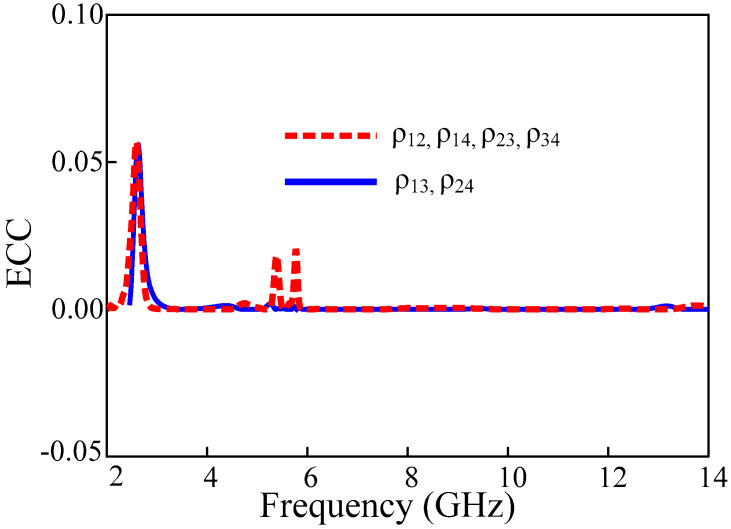
The envelope correlation coefficient of the proposed UWB-MIMO antenna.

The envelope correlation coefficient of the proposed UWB-MIMO antenna is shown in Figure 12. In MIMO systems the envelope correlation coefficient (ECC) expresses the independence of the single element performance, such as radiation pattern and polarization.

The ECC of MIMO can be calculated using S-parameters and far-field radiation patterns using Equations (1) and (2), respectively.

(1) $$\rho_{\mathrm{eij}}=\frac{{\left|S_{\mathrm{ii}}{*S}_{\mathrm{ij}}+S_{\mathrm{ji}}{*S}_{\mathrm{jj}}\right|}^{2}}{\left(1-{\left|S_{\mathrm{ii}}\right|}^{2}-S_{\mathrm{ij}}{}^{2}\right)\left(1-{\left|S_{\mathrm{ji}}\right|}^{2}-S_{\mathrm{jj}}{}^{2}\right)}\;$$

(2) $$\rho_{\mathrm{eij}}=\frac{{\left|\iint_{0}^{4\pi }\left[\vec{R_{i}}\left(\theta ,\;\varphi \right)\times \vec{R_{j}}\left(\theta ,\;\varphi \right)\;\right]d\Omega \right|}^{2}}{\iint_{0}^{4\pi }{\left|\vec{R_{i}}\left(\theta ,\;\varphi \right)\right|}^{2}d\Omega \iint_{0}^{4\pi }{\left|\vec{R_{j}}\left(\theta ,\;\varphi \right)\right|}^{2}d\Omega \;}\;$$

### 3.4. Diversity Gain

**Figure 13:**
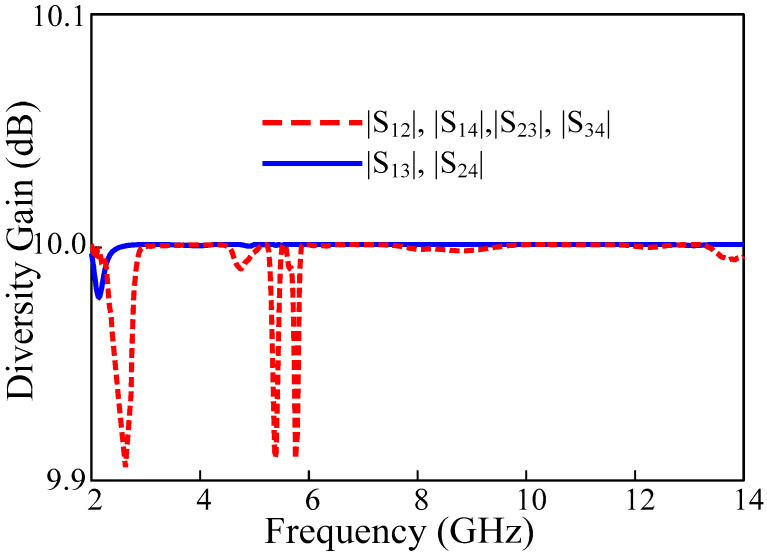
Diversity gain of the proposed UWB-MIMO antenna.

Using diversity gain, the effect of the diversity scheme on the radiated power can be described. Figure 13 shows the diversity gain of the proposed MIMO antenna which is a flat line in the ideal value for 10 dB for all four elements except the notched band. The DG is computed as a function frequency using relation (3).

(3) $$\mathrm{DG}=10\sqrt{1-{\left|\rho ij\right|}^{2}}\;$$

### 3.5. Channel Capacity Loss

Channel capacity is the data rate supported in a particular channel in a fading environment. The channel capacity loss is the maximum available transmission for which a signal can be transmitted over a particular MIMO antenna system. In practice, the value of CCL should be less than 0.4 bps/Hz. The capacity loss can be determined by using the following Equation (4):

(4) $$\mathrm{CCL}=-\log_{2}\det\left|\psi^{R}\right|$$

where ψR is the receiving antenna correlation matrix, which is given as:

(5) $$\psi R=\left[\begin{array}{c} \begin{array}{ccc} \rho 11 & \rho 12 & \rho 13\\ \rho 21 & \rho 22 & \rho 23\\ \rho 31 & \rho 32 & \rho 33 \end{array}\;\begin{array}{c} \rho 14\\ \rho 24\\ \rho 34 \end{array}\;\\ \rho 41\;\;\;\rho 42\;\;\;\rho 43\;\;\rho 44 \end{array}\right]\;$$

ρii = (1 − |Sii|^2^ − |Sij|^2^) and ρij = −(S∗iiSij + S∗jiSij) for i, j = 1 2, 3 or 4.

**Figure 14:**
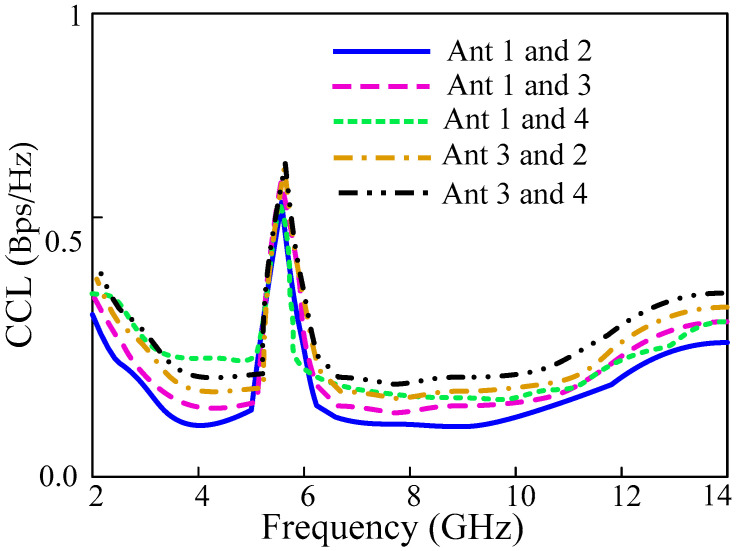
Chanel capacity loss analysis of the proposed UWB-MIMO antenna.

The CCL graph shown in Figure 14 demonstrates that the antenna CCL is than 0.4 bps/Hz for the entire UWB band except for the rectangularly notched WLAN band.

### 3.6. Mean Effective Gain

**Figure 15:**
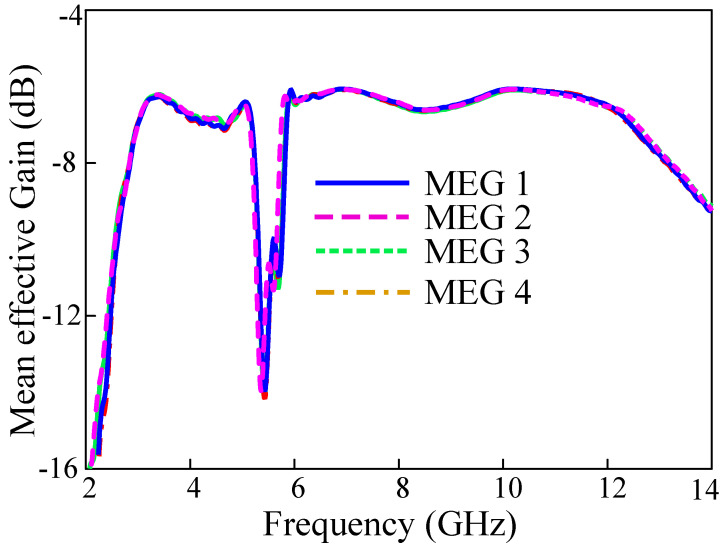
Variation of MEG with frequency.

The mean effective gain is the measure of receipt power levels of a MIMO antenna system to an isotropic radiator. The difference between MEG of ports of antenna always should be less than −3 dB. Equation (6) is used to calculate the MEG values:

(6) $$\mathrm{MEG}i=0.5\left(1-\overset{N}{\underset{i=1}{\sum }}\left|\mathrm{Sij}\right|\right)\;\;$$

where *i* represent the port under observation and *N* is the number of antennas in the MIMO system. The MEG of the proposed MIMO antenna is shown in Figure 15. The MEG values are very low (less than −12 dB) at the stop-band, while it is around −7 dB within the frequency range of interest.

### 3.7. Comparison with State-of-Artwork

The performance comparison of proposed rectangularly notched UWB-MIMO with stat-of-artwork is summarized and shown in Table 3.

The comparison is made with other UWB-MIMO antennas to the isolation enhancement technique, peak gain, and minimum isolation. The UWB−MIMO antenna [9] consists of two slot antennas, a T-shaped slot is etched on the ground to improve isolation. The antenna acquires a low mutual coupling of less than 18 dB in operating bandwidth. Whereas the references [10] are designed to use defected ground approach, that offers minimum isolation of 18 dB and peak gain of 4.9 dBi. Moreover, 2 port MIMO antennas reported in [11,12] have reached maximum isolation of 17 dB and 15 dB, respectively. Moreover, a 2 port UWB MIMO in [13] has shown a good isolation agreement of 17 dB and a peak gain of 3.5 dBi without any utilization of radiating elements. Furthermore, the MIMO antennas [14,15] contribute a customized parasitic decoupling structure on the backside of the antenna, which has an isolation of 20 dB and 15 dB correspondingly. Moreover, using neutralization lines, the mutual coupling is reduced up to 14.5 dB and peak gain of 5 in [13]. However, different UWB-MIMO antennas from [17,18,19,20] enhanced isolation using different-shaped stubs between single antenna elements. Finally, the paper [24] presents a UWB-MIMO antenna with the isolation of −20 dB using metamaterials. It is noted the antenna size is larger than the conventional antennas due to the presence of the EBGs, which have been used to realize the rectangular notch features.

The proposed antenna has better performance than the aforesaid antennas. This proposed antenna offers very high isolation and stable gain curve with a maximum gain of 6.94 dBi. Overall, our antenna is rectangularly notched and offers higher operating bandwidth while maintaining high gain and high isolation.

## 4. Conclusions

A rectangularly notched band UWB-MIMO antenna with an overall size of 60 × 60 × 1.52 mm^3^ is presented in this paper. Initially, the UWB antenna with a rectangular notching characteristic is designed. The notching characteristic is achieved by using two EBG structures. The proposed single-element is remodeled to a 2 × 2 UWB-MIMO system by translating each antenna element perpendicular to each other. The performance, particularly the isolation of the antenna is improved by using a novel parasitic structure. The simulated and measured results of the proposed antenna show a good agreement. The antenna offers a stable gain with a peak gain of 6.8 dBi, low isolation (12 dB improved compared without decoupling structure), low ECC value (0.001), and high diversity gain of 9.99. Hence the performance criteria indicate the stability of the proposed antenna for the UWB-MIMO application.

## Figures and Tables

**Table 1:** The optimized parameter of the proposed antenna.

Parameters	*s*_w_	*s*_l_	*h*	*p_w_*	*p_l_*	*l_g_*	*f*_w_	*w_d_*	*r*	*l*_3_	*l*_5_	*l*_6_
Units (mm)	60	60	1.5	15	12	7.4	4.1	0.9	0.3	20	10	4.1
Parameters	*w*_1_	*w*_2_	*l*_1_	*l*_2_	*s*	*s*_1_	*via*	*l*_4_	*r*_1_	*r*_2_	*l*_7_	*w*_3_
Units (mm)	1	1.8	15.2	15.2	3.5	11.3	0.2	20	6	5	2.9	3

**Table 2:** Simulated and measured gains of the MIMO antenna.

Freq (GHz)	Gain (dBi) of Antenna Element							
	Simulated Values				Measured Values			
	Port 1	Port 2	Port 3	Port 4	Port 1	Port 2	Port 3	Port 4
3	4.42	4.49	4.49	4.35	4.3	4.4	4.4	4.25
5	3.5	3.63	3.56	4.02	3.3	3.45	3.4	3.8
7	5.42	5.47	5.62	5.69	5.35	5.32	5.4	5.6
9	6.77	6.8	6.94	6.75	6	6.5	6.6	6.55

**Table 3:** Comparison of the proposed UWB-MIMO antenna with state-of-the-art works.

Ref.	Isolation Enhancement Technique	Antenna Size (mm^3^)	ECC	Minimum Isolation (dB)	Peak Gain (dBi)
[10]	Defected ground	29 × 40 × 0.508	0.0005	18	4.9
[11]	Defected ground	22 × 29 × 0.8	0.03	17	6
[12]	Decoupling element	30 × 40 × 0.8	0.05	15	4.2
[13]	Space diversity	25 × 50 × 1.6	0.43	17	3.5
[14]	Parasitic decoupler	40 × 43 × 1	0.2	20	4
[15]	Parasitic strip	34 × 34 × 1.6	0.05	15	5.5
[16]	Neutralization rings	75.19 × 75.19 × 1.6	0.1	14.5	5
[17]	F shaped stub	50 × 30 × 1.6	0.04	18	2.91
[18]	Used slots	15 × 25 × 1.6	0.02	20	NG
[19]	Inverted-Y shaped stub	58 × 45 × 1.6	0.02	15	NG
[21]	Inverted-Y shaped stub	33 × 38 × 1.6	0.08	17	6.2
[24]	Metamaterial	30 × 60 × 1.6	0.01	20	5.5
**This work**	**Parasitic decoupler**	**60 × 60 × 1.52**	**>0.001**	**21**	**6.94**

## Data Availability

Not applicable.
